# Analysis of microbial composition in different dry skin areas of Beijing women

**DOI:** 10.3389/fmicb.2025.1504054

**Published:** 2025-08-07

**Authors:** Jingtao Wang, Benyue Li, Yuanman Zhang, Wei Ma, Ting Jia, Jie Yang, Yexiang Zhang, Fengwei Qi, Yan Jia, Liya Song

**Affiliations:** ^1^Beijing Key Laboratory of Plant Resources Research and Development, Department of Cosmetics, School of Light Industry Science and Engineering, Beijing Technology and Business University, Beijing, China; ^2^Shandong Huahuitang Biotechnology Co., Ltd., Shanghai, China

**Keywords:** dry skin, women, skin microbiota, hand, lower leg

## Abstract

**Introduction:**

The composition of the skin microbiota is shaped by the interaction of multiple factors, with area-specific changes and physiological characteristics in the skin having the most profound impact. The back of the hand and lower leg are two dry areas of human skin. Whether their microbial compositions are consistent, as well as the changes in skin microbiota at these two areas among individuals with dry skin, warrant further discussion.

**Methods:**

Using 16S rRNA sequencing, we analyzed the differences of microbiota in dry skin areas of 54 young women and their changes in dry populations. Concurrently, key physiological parameters (Hydration, TEWL, sebum secretion) were measured.

**Results:**

Analysis of physiological parameters showed that Hydration, TEWL and sebum secretion were significantly lower (*P* < 0.05) in the lower leg compared to the back of the hand. Hydration was significantly lower (*P* < 0.05) at the same skin area in the dry-skinned population compared to the healthy population. Regarding microbial composition, the relative abundance of Firmicutes and Proteobacteria was significantly higher on the lower leg compared to the back of the hand, whereas the relative abundance of Actinobacteriota was notably greater on the back of the hand than on the lower leg (P < 0.05). Compared to the lower leg, the back of the hand showed a higher relative abundance of *Cutibacterium* (34.19% vs. 8.68%), whereas the lower leg was predominantly colonized by *Streptococcus* (17% vs. 13.76%). At the genus level, the relative abundance of *Streptococcus* was significantly increased in the dry skin group, whereas a decreasing trend was observed for *Cutibacterium*. Redundancy analysis (RDA) showed that Streptococcus was negatively correlated with Hydration, TEWL, and sebum, and vice versa for *Cutibacterium*.

**Dicussion:**

These findings suggest that differences in skin microbiota are primarily influenced by a combination of skin area micro environmental factors and not solely dependent on dryness status, suggesting that area-specific ecological niche design should be taken into account when conducting clinical interventions.

## 1 Introduction

The skin is the largest organ of the human body, serving as a dynamic interface between the body and the external environment, maintaining the integrity and functional stability of the body, and serving as the first line of defense against pathogens, ultraviolet radiation, and chemical and physical agents (Egawa et al., 2002; Kono et al., 2022). The most important function of the skin is its barrier function, which includes physical, chemical, immunological, and microbial barriers (Brettmann and Strong, 2018). The physical barrier, also known as the skin barrier, is primarily formed by the “brick and mortar” structure composed of stratum corneum cells (bricks) and intercellular lipids (mortar), which protects against external antigens, microbes, sunlight, and other irritants while preventing the loss of nutrients and water from the body (Madison, 2003; Nishifuji and Yoon, 2013). When the skin barrier is damaged, it can result in heightened water loss, leading to dry, flaky, and wrinkled skin, and potentially skin diseases (Cui et al., 2016). Depending on the water and oil characteristics of different skin areas, the skin is mainly divided into oily, moist, and dry areas (Boxberger et al., 2021). The back of the hand and the lower leg are dry areas of the body. These two areas with thicker stratum corneum are more prone to dryness and cracking due to the lack of sebum protection, and their skin barriers are more easily damaged, making it difficult to maintain bodily balance.

The skin is colonized by a variety of microorganisms, and the skin microbiota coexists with skin surface tissues, cells, and various secretions to form a microecosystem (Kanangat and Skaljic, 2021). Normally, the skin microbiota maintains a dynamic balance where microorganisms can produce antimicrobial peptides and short-chain fatty acids to serve as a barrier against pathogenic microorganisms (Szabo et al., 2017). In addition, recent studies have shown that the skin microbiota can be regulated through the aryl hydrocarbon receptor (AHR) signaling pathway in keratinocytes to promote epithelial differentiation and integrity, helping maintain the physical barrier function of the skin (Almoughrabie et al., 2023). Therefore, the skin microbiota is closely related to skin barrier and skin health. Dry areas are more susceptible to barrier damage and ecological disorders than other areas of the skin. For example, patients with atopic dermatitis (AD) exhibit barrier damage in affected areas. The abundance of *Staphylococcus aureus* is significantly increased in these areas and becomes progressively more prominent as the severity of the disease worsens (Paller et al., 2019; Zheng et al., 2019).

The composition of the microbial flora present on the skin differs according to its physiological environment. Changes in the density and quantity of sweat glands and hair follicles create completely different ecological niches for microbial growth (Otto, 2020). Studies have found that in damp areas such as armpits, the most abundant bacteria are *Staphylococcus* (Firmicutes) and *Corynebacteria* (Actinobacteria). Oily areas (face) have the most diverse populations with *Cutibacterium acnes* (Actinobacteria) being the most common isolate. Dry skin areas (legs and arms) show the highest diversity with Betaproteobacteria and Alpha proteobacteria being dominant (Byrd et al., 2018; Grice et al., 2009; Grice and Segre, 2011). Current comparative studies of the skin microbiota primarily concentrate on areas exhibiting notable disparities in physiological features. The skin on the back of the hand, the lower leg, and other areas typically have lower levels of moisture and oil, making them dry areas of the body’s skin. However, there are local differences in these dry areas, such as the thickness of the epidermis, mechanical strength, elasticity, degree of keratinization, types, and density of glands, pigmentation, and susceptibility to external influences (Chen et al., 2018; Sandby-Moller et al., 2003). In this study, we used high-throughput sequencing technology to analyze the microbial composition of dry skin areas such as the back of hand and the lower leg of young women in Beijing, China. We also compared the microbiota of healthy and dry populations in these two regions and revealed that skin microbial diversity is regulated by a combination of internal and external factors in addition to the skin area as an influencing factor. This finding provides an important ecological basis for the development of targeted skin care strategies.

## 2 Materials and methods

### 2.1 Instruments and materials

Sodium chloride (NaCl) and Tween 20 were purchased from Beijing Pharmaceutical Reagents Co., Ltd., China. Lysozyme was obtained from Beijing Baolingwei Chemical Technology Co., Ltd., China. DNeasy Blood and Tissue Kit was obtained from QIAGEN, Germany. Other materials included disposable sterile cotton swabs (Jiangsu Balance Medical Industries Co., Ltd., Jiangsu, China). The Multi Probe Adapter (MPA9) multifunctional skin testing instrument was from Courage+Khazaka, Germany.

### 2.2 Subjects and inclusion criteria

The participants were recruited from female individuals in Beijing aged 18–29 years. Within seven days before sampling, the participants should not have applied any antibiotics or hormone-based products on their skin surfaces. They should not have taken any antibiotics or antibacterial drugs orally within the past month. In addition, participants must be excluded if they have a history of eczema, acne, or other medical conditions. If the skin at the test area exhibits birthmarks, pigmentation, inflammation, scars, pigmented nevi, or excessive hair, such cases should also be excluded. A total of 54 participants were selected through questionnaires designed and distributed according to internationally recognized drying grade criteria and research objectives. This study has been approved by the Ethics Committee of Beijing Technology and Business University and obtained written informed consent from all research participants. The study was conducted in accordance with the ethical rules for human experimentation stipulated in the Helsinki Declaration.

### 2.3 Skin physiology parameter testing

All participants were required to wash the test area with clean water after microbial collection on the measurement day and remain seated for 30 min in a constant temperature and humidity environment (21 ± 1°C and 50 ± 5%). And use the German CK Skin analyzer (MPA580) Corneometer CM825 (moisture test probe), Tewameter TM300 (moisture loss test probe), Sebumeter (oil test probe), Skin-pH-Meter PH905 (pH test probe), MPA580 (skin elasticity test probe), Skin moisture content (Hydration), transdermal water loss (TEWL), skin sebum content, skin pH, and skin elasticity (R2) in the 2 × 2 cm^2^ area of the back of the hand and the outer side of the lower leg, respectively.

### 2.4 Microbial sample collection

In September 2023, the sample collection from participants was carried out. Prior to collection, participants were required to refrain from facial cleansing or applying any cosmetics for 8 h. Before collection, participants sat quietly for 20 min in a constant temperature and humidity environment (21 ± 1°C and 50 ± 5%) to stabilize their condition before microbial samples were collected. During the sampling process, the researchers took two sterile cotton swabs dipped in moistening solution (0.9% NaCl - 0.1% Tween 20) and then used to wipe the back of the hand and the outer side of the lower leg in a 4 × 4 cm^2^ area of skin 50 times each. The swabs are then placed into collection tubes, and the samples are stored in dry ice, subsequently transferred to −80°C storage, and DNA extraction is performed as soon as possible. The extracted DNA was sent to Shanghai Meiji Biomedical Co., Ltd. for sequencing.

### 2.5 DNA extraction and sequencing

In this study, the DNeasy Blood and Tissue Kit from QIAGEN was used to extract total DNA from the surface bacteria of the skin. The specific steps were referenced from previously published literature by our research group. The V1–V3 variable region was amplified by PCR using the primers 27F (5′-AGAGTTTGATCCTGGCTCAG-3′) and 533R (5′-TTACCGCGGCTGCTGGCAC-3′). PCR reaction conditions: pre denaturation at 95°C for 3 min; Denatured at 95°C for 30 s, annealed at 55°C for 45 s, extended at 72°C for 45 s, extended at 72°C for 10 min after 35 cycles, and then kept at 10°C. Illumina Miseq sequencing was performed according to methods previously published by our research group (Zheng et al., 2019).

### 2.6 Processing of sequencing data

Raw FASTQ files were de-multiplexed using an in-house perl script, and then quality-filtered by fastp version 0.19.6 and merged by FLASH version 1.2.7. Using UPARSE v7.1 software^1^, operational taxonomic unit (OTU) clustering was performed on quality-controlled and assembled sequences at 97% similarity threshold, followed by chimera removal. Sequences annotated as chloroplast or mitochondrial origins were eliminated from all samples. To minimize the impact of sequencing depth on subsequent alpha and beta diversity analyses, all sample sequences were rarefied to 20,000 reads (sequence rarefaction is recommended). After rarefaction, the average sequence coverage (Good’s coverage) for each sample remained at 99.09%. Using the RDP classifier^2^, taxonomic annotation of OTUs was performed by aligning with the Silva 16S rRNA gene database (v138) at a confidence threshold of 70%. The community composition of each sample was then statistically analyzed across different taxonomic levels.

### 2.7 Statistical analysis

Using GraphPad Prism 9.5 software, we performed *t*-tests on skin physiological parameters to analyze differences between different groups. Using the mothur software, we calculated the Chao1 index and Shannon index to evaluate α-diversity. Principal coordinate analysis (PCoA) based on the Bray-Curtis distance algorithm was employed to investigate the similarity of microbial community structures among samples, while analysis of similarities (ANOSIM) was used to determine significant differences in β-diversity of microbial communities. Non-parametric Wilcoxon signed-rank test and Wilcoxon rank-sum test were applied to compare microbial diversity differences between groups. MaAslin2 was utilized to examine the associations between microorganisms at the genus level and skin sites/skin conditions. Distance-based redundancy analysis (db-RDA) was conducted to explore the influence of skin physiological parameters on microbial community structure. Based on Spearman correlation analysis, we selected species with |r| > 0.6 and *p* < 0.05 for correlation network analysis, and visualization was performed using the pheatmap package in R (version 3.3.1). Single-factor networks were constructed by calculating correlations between species to generate species correlation networks. All data analyses were completed using the free online Majorbio I-Sanger Cloud Platform.^3^

## 3 Results

### 3.1 Bacterial composition of different dry skin areas

#### 3.1.1 Analysis of skin physiological parameters results

**FIGURE 1:**
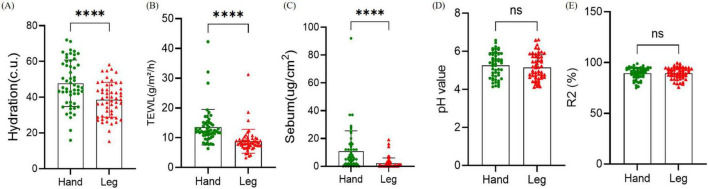
Comparison of physiological parameters between the back of the hand and the lower leg. **(A–E)** Hydration, transepidermal water loss (TEWL), sebum content, pH, and skin elasticity. Difference test: Paired *t*-test is adopted, ns means *P* ≥ 0.05, ****means *P* ≤ 0.0001.

Physiological parameters of different areas of women’s dry skin were evaluated. The skin physiological parameters of 54 volunteers were measured on the skin of the back of the hand and the lower leg (Figure 1). Skin moisture content (Hydration), transepidermal water loss (TEWL) and sebum content showed significant differences in the two different skin areas of the back of the hand and the lower leg (*P* < 0.001). Hydration, TEWL, and sebum content in the lower leg were significantly decreased. There were no significant differences in skin pH and skin elasticity between the two dry areas (*P* > 0.05).

#### 3.1.2 Inter-group bacterial diversity analysis

**FIGURE 2:**
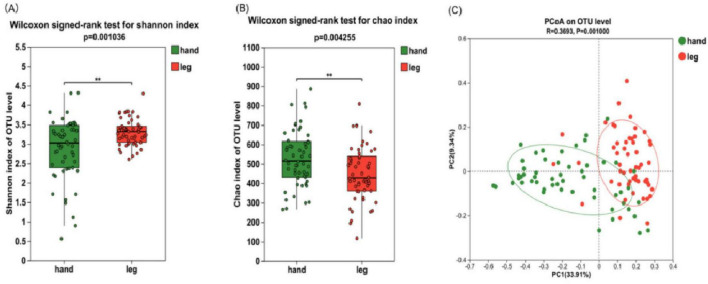
Microbial diversity in the skin of the back of the hand and the lower leg. **(A)** Shannon index; **(B)** Chao index; **(C)** principal coordinate analysis (PCoA) at the operational taxonomic unit (OTU) level. Difference test: Wilcoxon signed-rank test was used, where **means *P* ≤ 0.01.

To elucidate the changes in Alpha diversity of two groups of bacterial communities, the Chao richness values and Shannon diversity values of the two groups were compared (Figures 2A, B). Compared with the back of the hand, Shannon index of the lower leg was significantly increased (*P* < 0.01) and Chao index was significantly decreased (*P* < 0.01), indicating that the species diversity of the lower leg skin was significantly higher than that of the back of the hand, while the species richness was significantly lower than that of the back of the hand. Principal Coordinate Analysis (PCoA) of bray-curtis based on microbiota compositional profiles at the OTU level showed that the bacterial composition of the two dry skin areas, the back of the hand and the lower leg, was significantly different, indicating that the species composition and relative abundance of different skin areas were different (Figure 2C, *P* ≤ 0.001).

#### 3.1.3 Taxonomic profiling and differential abundance analysis of skin bacteria

A total of 8,795,980 sequences were obtained from 108 bacterial samples from 54 volunteers, with sequence lengths ranging from 441 to 500 bp and an average sequence length of 475 sequences. By clustering and annotation at a 97% similarity level, 6,020 OTUs were obtained, belonging to 39 phyla, 1266 genera, and 2,862 species.

**FIGURE 3:**
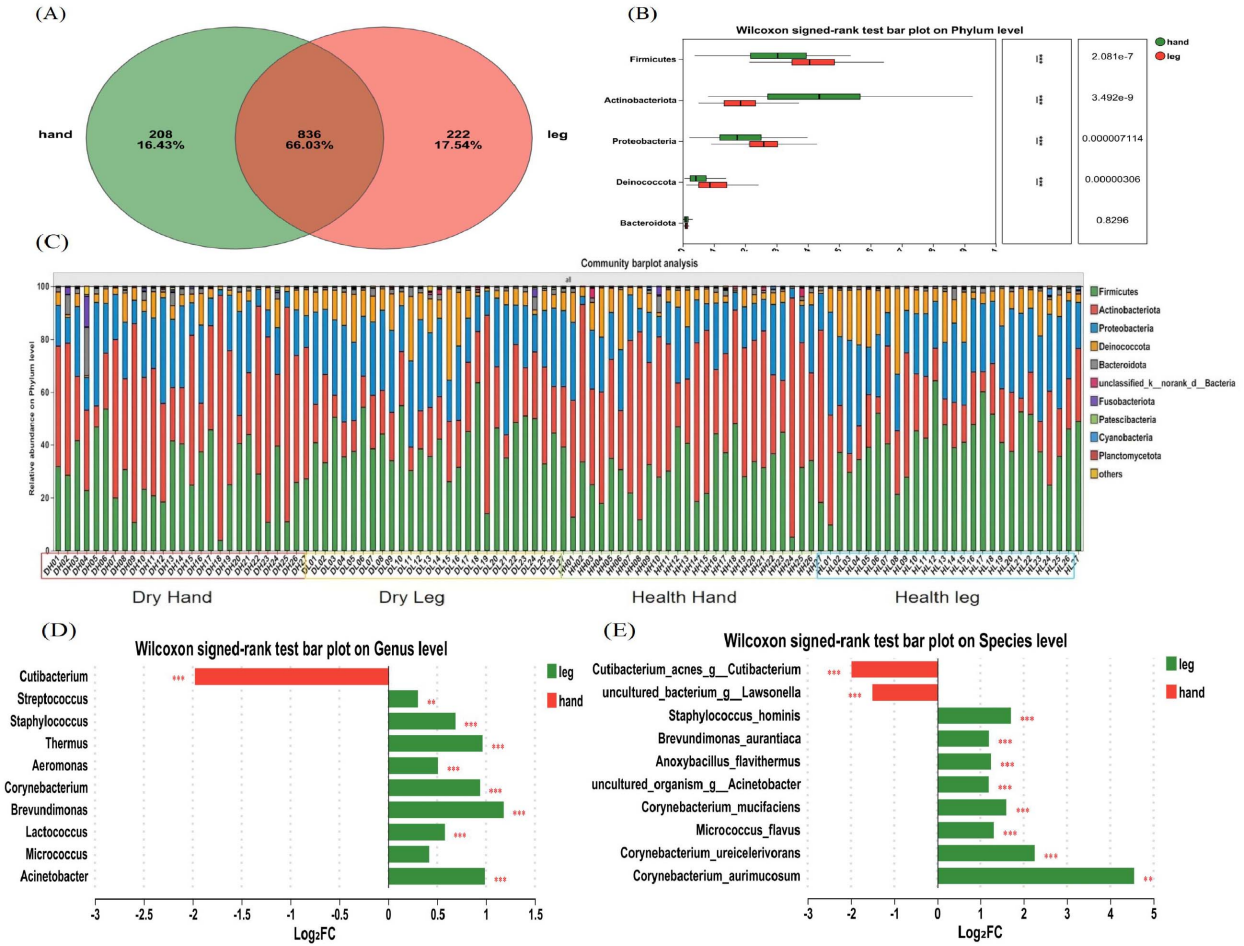
Composition and diversity analysis of bacterial communities. **(A)** A Venn diagram at the OTU level; **(B)** Differences in microbial composition at the phylum level; **(C)** Microbial composition of all samples at the phylum level; **(D)** Differences in microbial composition at the genus level. **(E)** Differences in microbial composition at the species level. Difference test: Wilcoxon signed-rank test was used, where **means P ≤ 0.01, and ***means P ≤ 0.001.

To study the microbiota structure at different dry skin areas, differences in composition between phylum, genus, and species levels were analyzed in hand back and lower leg samples. The Venn diagram (Figure 3A) shows the number of shared and unique OTUs between the back of the hand and the lower leg, showing that 43.12% of the OTUs are shared between the two groups, 29.37% are unique to the back of the hand, and 27.51% are unique to the lower leg. At the phylum level, the bacteria in all skin samples were mainly composed of Firmicutes, Actinobacteria, Proteobacteria, and Deinococcota, accounting for more than 95% of the total bacteria (Figure 3C). The results of microbial differential analysis at the phylum level (Figure 3B) showed significant differences in the main bacterial components (*P* < 0.001). Except for Actinobacteria, the other three dominant bacteria had a higher proportion in the lower leg. At the genus level, the bacterial composition on the skin of the back of the hand and lower leg mainly consists of *Cutibacterium* (34.19%, 8.682%), *Streptococcus* (13.76%, 17%), *Staphylococcus* (9.616%, 15.48%), *Thermus* (5.311%, 10.36%), *Aeromonas* (5.065%, 7.206%), and *Corynebacterium* (3.434%, 6.581%). The results of the statistical table of difference test (Table 1) showed that except *Micrococcus* and *Kocuria*, other bacteria have significant differences in the back of the hand and the lower leg (*P* < 0.05). The relative abundance of *Cutibacterium* and *Pseudomonas* etc. in the back of the hand is significantly higher than that in the lower leg. *Streptococcus*, *Staphylococcus*, *Thermus*, and *Aeromonas* exhibited significantly greater relative abundances in the lower leg compared to the back of the hand (Figure 3D). At the species level, the bacterial species with relatively high abundance on the back of the hand and lower leg skin include: *Cutibacterium acnes*, *Streptococcus gallolyticus*, *Thermus scotoductus*, *Staphylococcus hominis*, and others. Based on the analysis results of microbial composition differences at the species level (Figure 3E), *Cutibacterium acnes* and *Staphylococcus sp.* have a high proportion on the back of the hand. *Streptococcus gallolyticus*, *Thermus scotoductus*, *Staphylococcus hominis*, etc., accounted for a high proportion in the lower leg.

**TABLE 1:** Difference analysis of dominant bacteria genera on the back of hand and calf (average abundance and top 20).

Species name	Hand mean (%)	Hand Sd (%)	Leg mean (%)	Leg Sd (%)	*P*-value	FDR *p*-value
*Cutibacterium*	34.19	22.27	8.682	7.773	4.81E-10	6.084E-7
*Streptococcus*	13.76	7.978	17	6.089	0.006021	0.1134
*Staphylococcus*	9.616	6.821	15.48	10.69	0.0001928	0.009035
*Thermus*	5.311	4.432	10.36	7.404	1.62E-6	0.0002771
*Aeromonas*	5.065	3.21	7.206	2.731	0.0001273	0.007003
*Corynebacterium*	3.434	4.692	6.581	7.439	0.0001464	0.007716
*Brevundimonas*	1.877	2.273	4.253	4.025	4.348E-8	2.75E-5
*Lactococcus*	1.847	1.27	2.754	1.014	4.007E-5	0.002534
*Micrococcus*	1.792	3.582	2.393	4.193	0.1019	0.6793
*Acinetobacter*	1.354	0.8602	2.686	3.24	1.804E-5	0.001475
*Klebsiella*	1.661	1.1	2.288	0.8991	0.001937	0.04948
*Pseudomonas*	1.673	3.801	1.578	1.246	0.006683	0.1208
*Escherichia-Shigella*	1.115	0.7813	1.589	0.6436	0.0006302	0.02171
*Anoxybacillus*	0.781	0.6916	1.844	2.001	3.947E-7	0.0001664
*Moraxella*	0.7094	1.538	1.102	2.239	0.0138	0.2103
*Paenibacillus*	0.5208	0.4588	1.162	1.097	7.557E-6	0.0008127
*Lactobacillus*	0.9196	3.636	0.4365	0.5672	0.167	0.8948
*Lawsonella*	0.9992	1.505	0.352	0.5506	3.928E-6	0.0004969
*Brevibacterium*	0.6708	2.163	0.5555	0.702	0.01963	0.2729
*Kocuria*	0.528	1.323	0.5456	0.77	0.007901	0.1334

### 3.2 Differential analysis of hand and leg skin microbiota in dry skin versus healthy cohorts

Based on participants’ self-assessed skin dryness conditions, they were divided into a dry skin group and a healthy control group. Participants in the “dry skin group” were further screened according to internationally recognized dryness grading criteria, with stratum corneum moisture content ≤ 40 AU; The “healthy control group” had no history of skin diseases, with stratum corneum moisture content ≥ 40 AU. The specific number of individuals in each group is shown in Table 2.

**TABLE 2:** Grouping.

Group			Number of people
Dry skin group	Hand	DH	17
	Leg	DL	
Healthy control group	Hand	HH	17
	Leg	HL	

#### 3.2.1 Analysis of skin physiological parameters results

**FIGURE 4:**
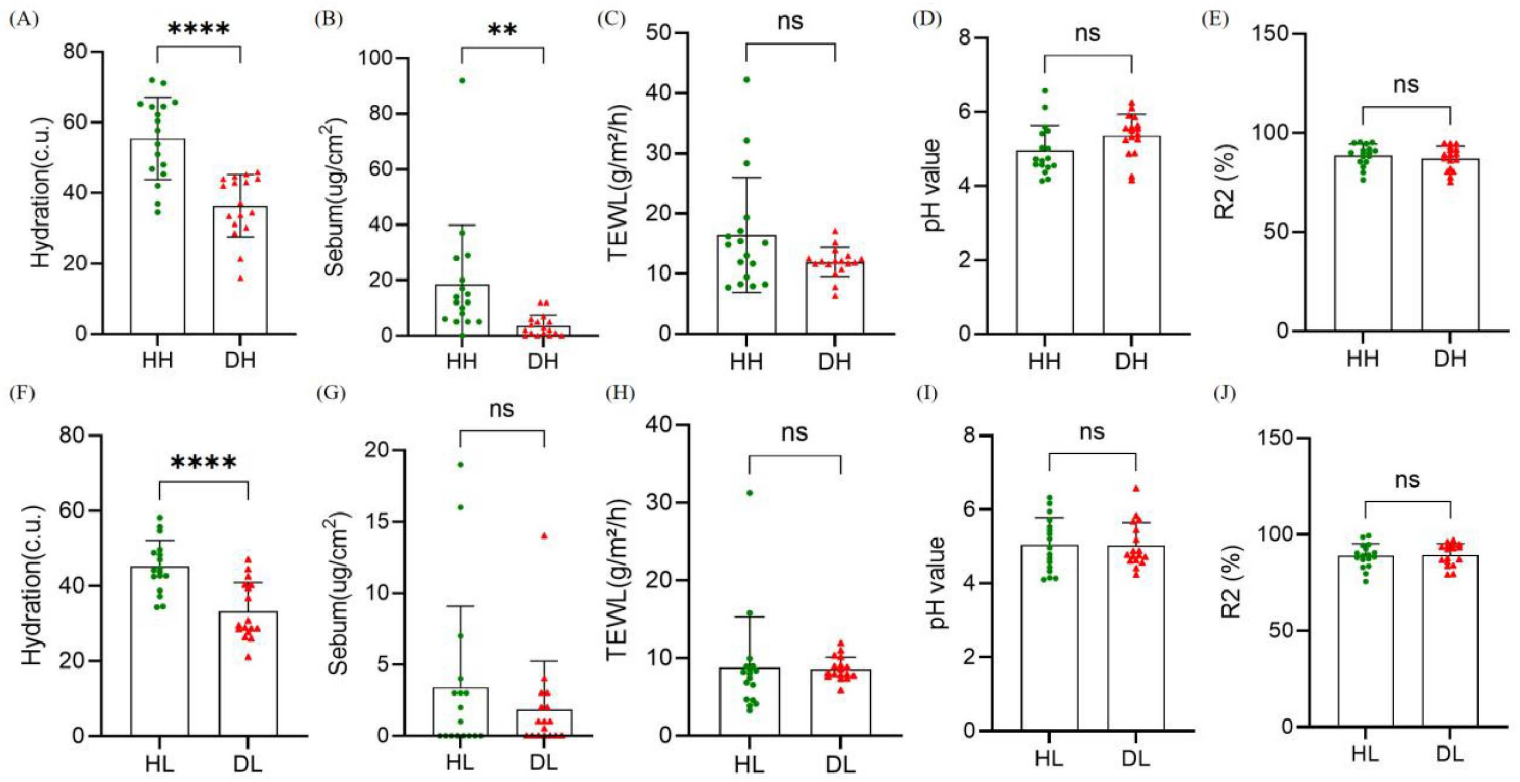
Comparison of skin physiological parameters between healthy group and dry group. **(A–E)** Hydration, transdermal water loss (TEWL), sebum content, pH and R2 in the back of the hand. **(F–J)** Hydration, TEWL, sebum content, pH, and R2 in the lower leg. Difference test: *t*-test is adopted, ns means *P* ≥ 0.05, **means *P* ≤ 0.01, ****means *P* ≤ 0.0001.

Comparison of skin physiological parameters between the dry skin group and the healthy control group in two skin areas (Figure 4). In the skin areas on the back of the hand, Hydration (*P* < 0.001) and sebum content (*P* < 0.01) in the dry skin group were significantly decreased compared with the healthy control group, and there were no significant differences in TEWL and other indicators (*P* > 0.05). In the skin area of the lower leg, the water content of the stratum corneum in the dry skin group was significantly reduced (*P* < 0.001), and there was no significant difference in the other indicators (*P* > 0.05).

#### 3.2.2 Differences in the microbial composition of the skin on the back of the hand

**FIGURE 5:**
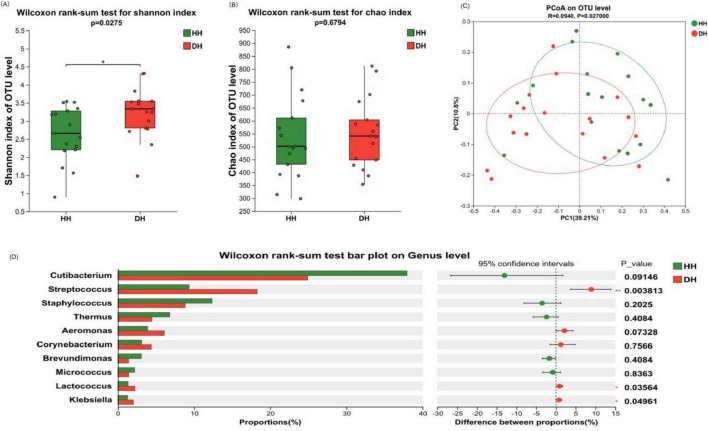
Composition and diversity analysis of bacterial communities on the back of the hand. **(A)** Shannon index. **(B)** Chao index. **(C)** Principal coordinate analysis (PCoA) at operational taxonomic unit (OTU) level. **(D)** Analysis of differences between the average abundance and the top 10 dominant bacteria genera. Difference test: Wilcoxon rank sum test was used, where *means 0.01 < *P* ≤ 0.05, and **means *P* ≤ 0.01.

According to the Alpha diversity analysis of bacteria on the back of hand skin, compared with healthy control group, the Shannon index in the dry skin group was significantly increased (*P* < 0.05), while the Chao index had no significant change (*P* > 0.05). Therefore, the back of hand skin in the dry skin group had more diverse species than that in the healthy control group, but the richness was similar (Figures 5A, B). The community composition of the healthy control group and the dry skin group was analyzed by principal coordinate analysis, and the microbiota of the healthy control group and the dry skin group were different (Figure 5C, *P* < 0.05).

The difference in bacterial composition between the healthy control group and the dry skin group was analyzed at different classification levels. At the phylum level, there was no significant difference between the healthy control group and the dry skin group in the four main phyla (see Supplementary Figure 1A). At the genus level, significant differences were observed in the relative abundances of *Streptococcus*, *Lactococcus*, and *Klebsiella* (*P* < 0.05), with the dry skin group exhibiting markedly higher proportions compared to the healthy control group. *Cutibacterium* also showed a significant decrease trend in the dry skin group (*P* < 0.1) (Figure 5D).

#### 3.2.3 Differences in the microbial composition of the skin on the lower legs

**FIGURE 6:**
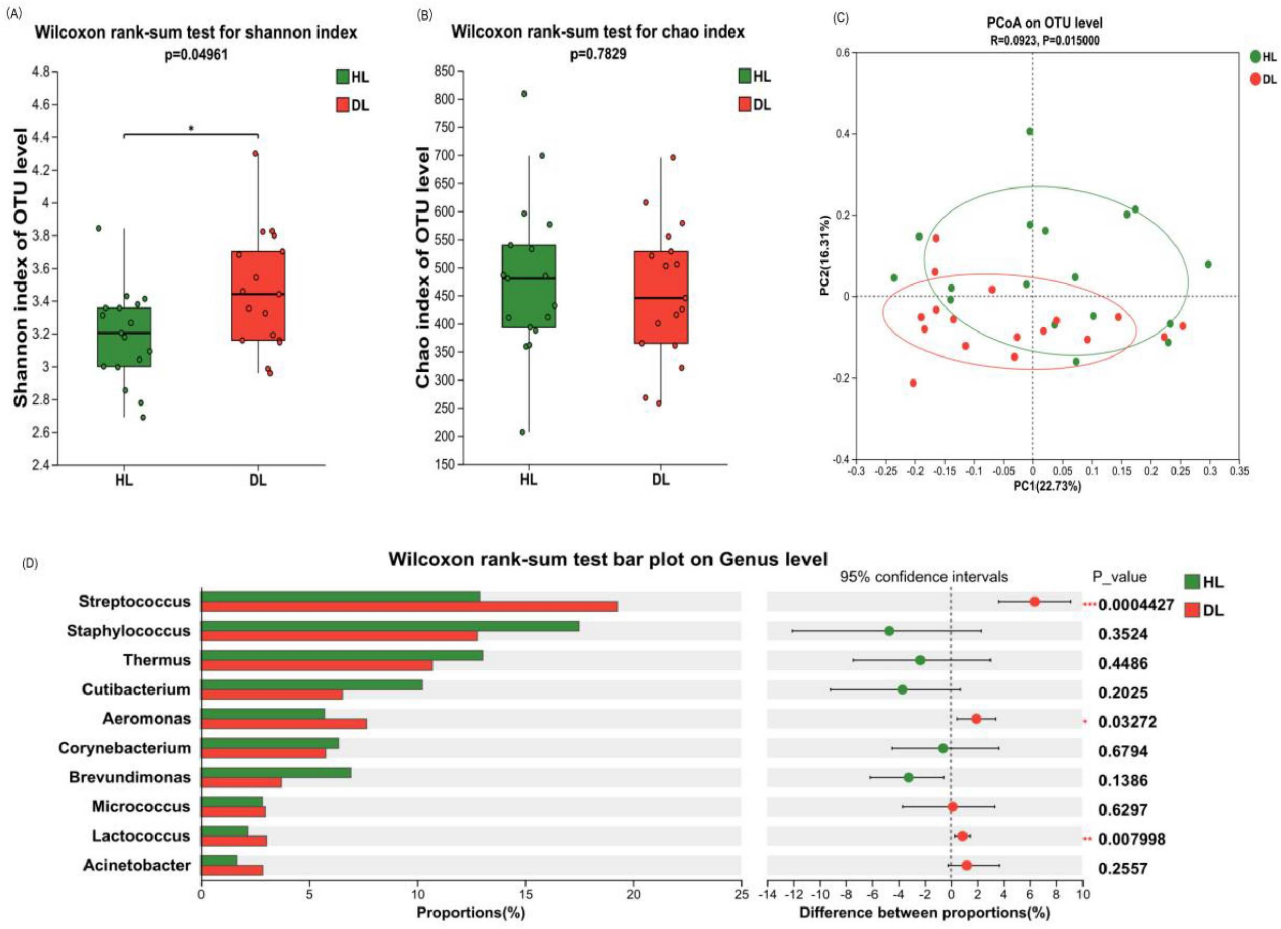
Composition and diversity analysis of crus bacterial communities. **(A)** Shannon index. **(B)** Chao index. **(C)** Principal component analysis (PCA) at operational taxonomic unit (out) level. **(D)** Analysis of differences between the average abundance and the top 10 dominant bacteria genera. Difference test: Wilcoxon rank sum test was used, where *means 0.01 < *P* ≤ 0.05, **means *P* ≤ 0.01, and ***means *P* ≤ 0.001.

Through the analysis of the Alpha diversity of bacteria on the skin of the lower leg, it was found that compared with the healthy control group, the Shannon index of the dry skin group was significantly increased (*P* < 0.05), while the Chao index was not significantly changed (*P* > 0.05). Therefore, the skin on the lower leg of the dry skin group had more diverse species than that of the healthy control group, but the richness was similar (Figures 6A, B). Beta diversity analysis via principal coordinate analysis (PCoA) demonstrated distinct clustering of microbiota between healthy controls and the dry skin cohort (Figure 6C, *P* < 0.05).

The difference in bacterial composition between the healthy control group and the dry skin group was analyzed at different classification levels. At the phylum level, there was no significant difference between the healthy control group and the dry skin group in the four main phyla (see Supplementary Figure 1B). At the genus level, *Streptococcus*, *Aeromonas*, and *Lactococcus* exhibited significantly elevated relative abundances in the dry skin group compared to healthy controls group (*P* < 0.05). The relative abundance of *Cutibacterium* on the lower leg did not show any difference between dry and healthy skin (Figure 6D, *P* = 0.2025).

### 3.3 Skin area and skin condition regulate microbial relative abundance: MaAsLin2 analysis

**FIGURE 7:**
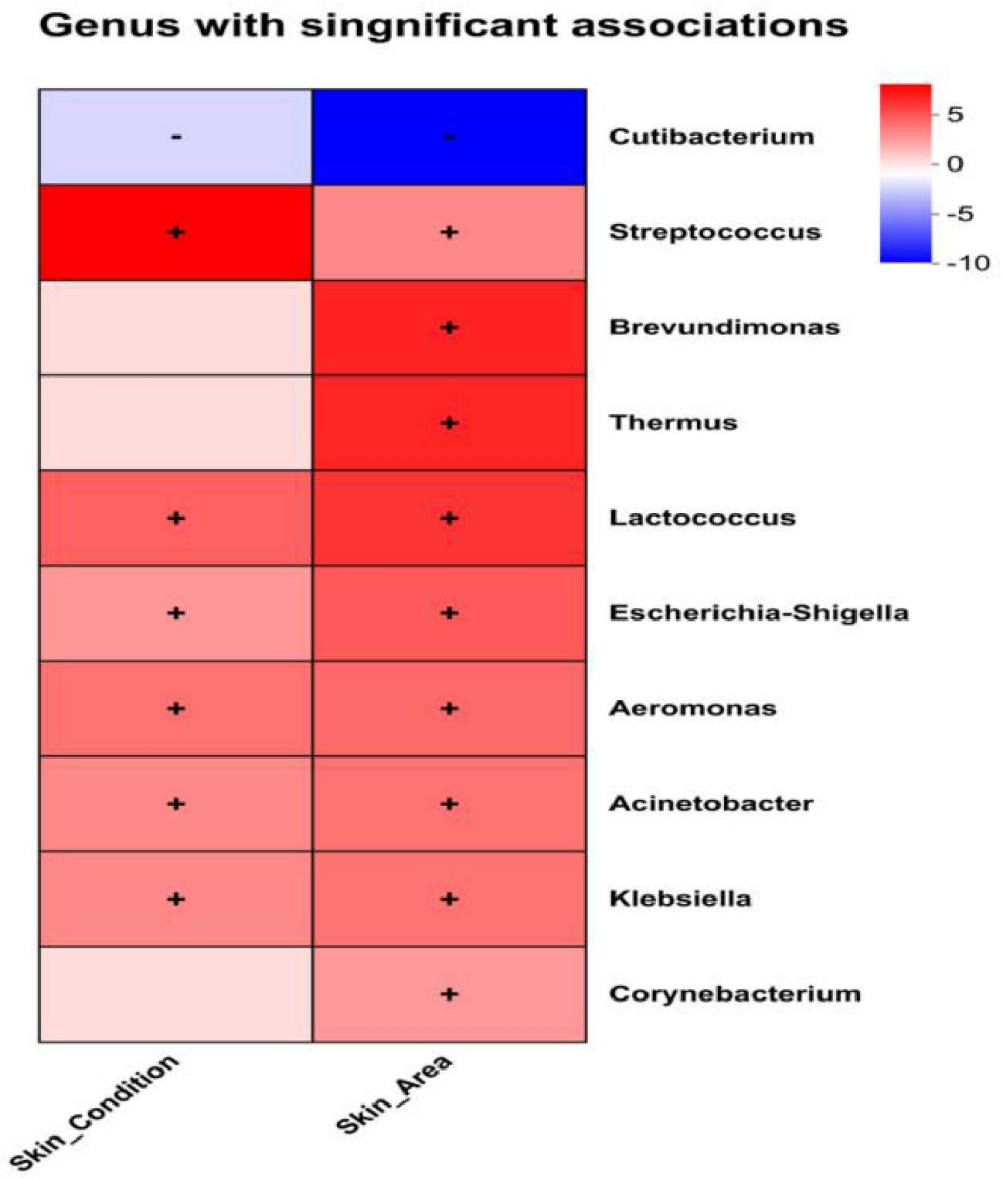
MaAsLin2 heatmap analysis; Significance threshold: *q*-value < 0.25, where “+” in the figure represents positive correlation and “–” represents negative correlation.

Analysis using MaAsLin2’s multivariate generalized linear mixed-effects model revealed associations between volunteer microbiome data and skin area (Skin Area) as well as Skin Condition (Table 3). The correlations were visualized via heatmaps, intuitively presenting the relationships between dominant bacterial genera and these variables (Figure 7). Skin Area (Hand vs. Leg) showed a significant negative correlation with *Cutibacterium* relative abundance (β = −1.855, *q* = 0.0001), indicating higher abundance of this genus in hand samples. Simultaneously, we observed significant positive correlations between Skin Area (Hand vs. Leg) and the abundances of *Brevundimonas* (β = +1.607, *q* = 0.003), *Thermus* (β = +1.340, *q* = 0.003), *Lactococcus* (β = +0.830, *q* = 0.005), *Escherichia Shigella* (β = +0.830, *q* = 0.005), and *Aeromonas* (β = +0.645, *q* = 0.028). In contrast, Skin Condition (Healthy vs. Dry) showed a significant positive correlation with the relative abundances of *Streptococcus* (β = +0.912, *q* = 0.001), *Lactococcus* (β = +0.701, *q* = 0.021), *Escherichia Shigella* (β = +0.701, *q* = 0.021), and *Aeromonas* (β = +0.618, *q* = 0.037). The study systematically validated the specific effects of Skin Area and Skin Condition on skin microbiome composition, supporting the results of inter-microbiome differential analysis from previous chapters.

**TABLE 3:** MaAsLin2 analysis significance correlation results table.

Feature	Metadata	β (coefficient)	*N* not 0	*P*-value	FDR *q*-value
*Cutibacterium*	Skin area	−1.85547	68	1.809742e-7	0.000124
	Skin condition	−0.79169	68	0.015243	0.093364
*Streptococcus*	Skin area	0.475	68	0.011087	0.076687
	Skin condition	0.9123	68	0.000004	0.001091
*Brevundimonas*	Skin area	1.60689	68	0.000032	0.003253
	Skin condition	–	68	–	> 0.25
*Thermus*	Skin area	1.34003	68	0.000066	0.003479
	Skin condition	–	68	–	> 0.25
*Lactococcus*	Skin area	0.82994	68	0.00012	0.005473
	Skin condition	0.7012	68	0.000963	0.021435
*Escherichia Shigella*	Skin area	0.82994	68	0.00012	0.005473
	Skin condition	0.7012	68	0.000963	0.021435
*Aeromonas*	Skin area	0.6452	68	0.001921	0.028036
	Skin condition	0.61765	68	0.002896	0.037128
*Acinetobacter*	Skin area	0.72486	68	0.002923	0.037128
	Skin condition	0.63555	68	0.008559	0.073391
*Klebsiella*	Skin area	0.62559	68	0.003285	0.038432
	Skin condition	0.55462	68	0.008681	0.073518
*Corynebacterium*	Skin area	0.80145	68	0.025272	0.127231
	Skin condition	–	68	–	> 0.25

This table presents results with significant differences where *P*-value < 0.05. Column *N* represents the total number of samples, and Column *N* not 0 represents the number of samples with non-zero species abundance.

### 3.4 The relationship between skin microorganisms and skin physiological parameters

**FIGURE 8:**
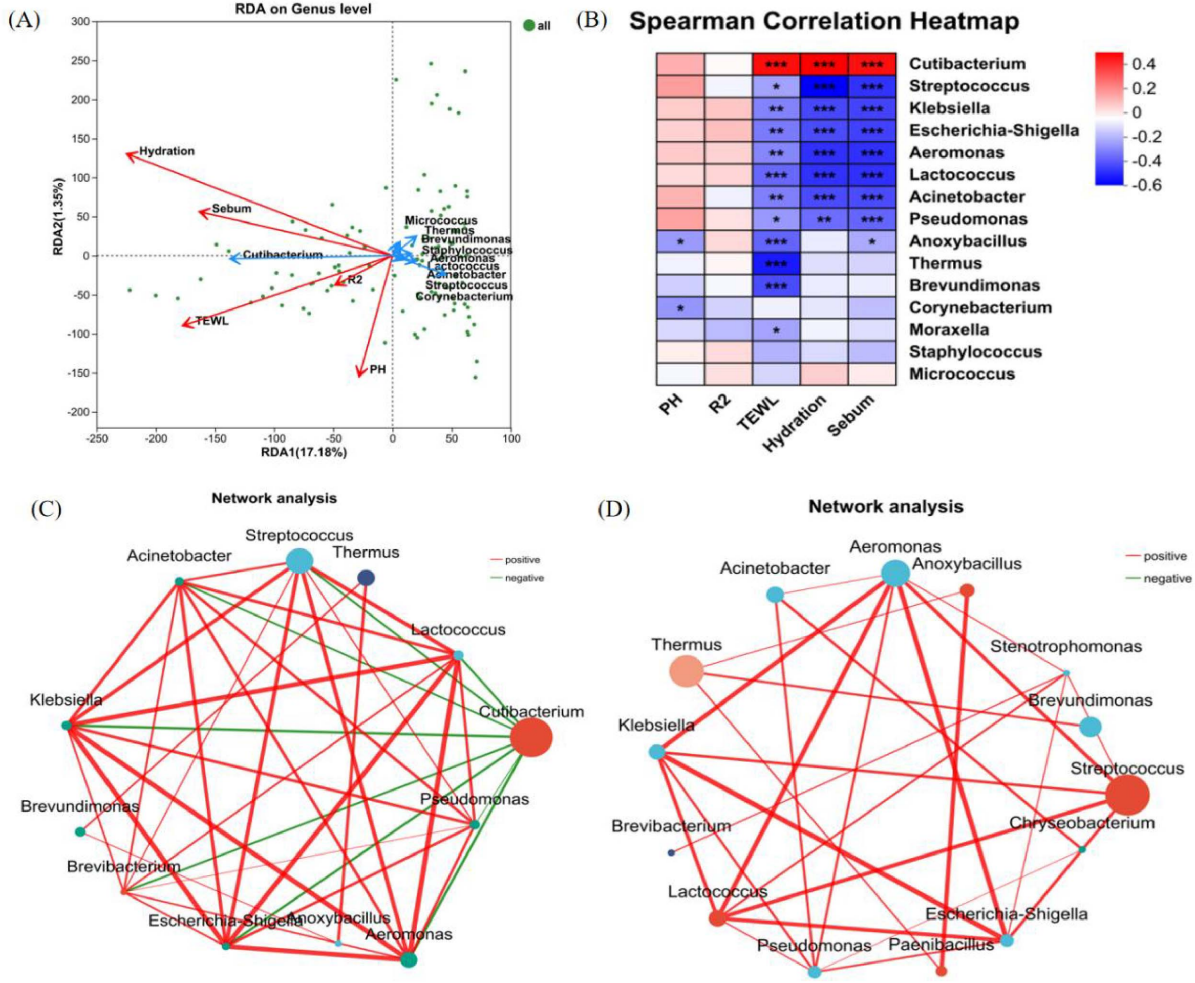
Correlation analysis. **(A)** RDA/CAA analysis reveals the relationship between relative abundance of bacterial genera and physiological parameters of the skin; (the length of the arrow of the physiological parameters can represent the extent to which it affects the species data). **(B)** Heat map of correlations between the top 15 bacterial genera in terms of relative abundance and physiological parameters of the skin; (R-value is shown in different colors in the figure, *means 0.01 < *P* ≤ 0.05, **means *P* ≤ 0.01, and ***means *P* ≤ 0.001). **(C)** Single-factor correlation network of the back of hand samples at the genus level. **(D)** Single-factor correlation network of the lower leg samples at the genus level (Only correlations with *P*-values < 0.05 are shown, node size represents the abundance level of species., the color grading of lines represents R-values, negative correlations are shown in green, and positive correlations are shown in red).

Redundancy analysis can analyze the degree of influence of skin physiological parameters on the composition of microbiota (Figure 8A). Hydration, TEWL, sebum content, and pH exhibited stronger associations with the overall structure of microbiota (based on genus-level relative abundances), while skin elasticity (R2) shows a weaker correlation. We used ggpubr in R to conduct regression analysis to determine the relationship between skin microbiota and skin parameters (R > 0.3). Correlation between skin biometric parameters and major bacteria genera showed that the relative abundance of *Cutibacterium* was positively correlated with Hydration (r = 0.45384, *P* < 0.001), TEWL (r = 0.42612, *P* < 0.001), and sebum (r = 0.42278, *P* < 0.001). Other dominant bacteria, including the relative abundance of *Streptococcus*, were significantly and negatively correlated with hydration (r = −0.52734, *P* < 0.001), TEWL (r = −0.21546, *P* < 0.05), and sebum content (r = −0.42844, *P* < 0.001). However, most of the relative abundance of bacteria showed no correlation with pH and skin elasticity (R2) (Figure 8B).

Inter-genus network correlation analysis revealed significant differences in bacterial structural frameworks between the back of the hand and the lower leg (Figures 8C, D). The skin microbial networks of the back of the hand and the lower leg were centered around *Cutibacterium* and *Streptococcus* as core nodes, respectively, forming extensive connections with multiple microorganisms. This result reflects the uniqueness of interaction patterns among microbes at different skin areas. In the microbial networks of both the back of the hand and the lower leg, genera such as *Streptococcus*, *Escherichia-Shigella*, *Klebsiella*, *Aeromonas*, and *Lactococcus* were closely associated through red connecting lines, suggesting potential mutualistic relationships in metabolism or ecological niches. Notably, negative correlations were observed between *Cutibacterium* on the back of the hand and genera such as *Streptococcus*, *Thermus*, and *Aeromonas*.

## 4 Discussion

The skin serves as a crucial barrier for the body to defend against the external environment and maintain internal homeostasis, with its structural integrity being essential for preventing water loss and external stimuli (Smith, 1999). A healthy skin barrier is like a tight “brick wall.” Once the structure becomes damaged, it disintegrates, leading to a deficiency in the skin’s protective capacity. Consequently, the skin is unable to effectively retain moisture internally, rendering it vulnerable to external irritants. This susceptibility gives rise to a range of issues, including skin dryness, among others (Fluhr et al., 2024). Dry skin (such as on the lower legs, back of hands, and other areas) is more susceptible to damage due to its fragile barrier function (Lange-Asschenfeldt et al., 2011). Therefore, this study conducted a comparative analysis of the two dry skin areas on the back of the hands and lower legs between healthy individuals and those with dry skin. Physiological parameter analysis revealed that the moisture content of the stratum corneum, TEWL, and sebum secretion in the lower leg were significantly lower than those on the back of the hand, indicating that the lower leg exhibited a higher degree of skin dryness. Compared to healthy individuals, the moisture content in the same skin area of individuals with dry skin was significantly reduced, while TEWL and pH showed no significant changes. Among the dry skin group, sebum secretion on the dorsum of the hand was significantly decreased, whereas no significant change was observed in sebum secretion on the lower leg. TEWL is an important means for clinical assessment of skin permeability barrier function (Sato et al., 2002). Akdeniz et al. (2018) proved that higher TEWL is generally associated with skin barrier damage and lower TEWL is associated with healthy skin by measuring 82 skin areas in subjects aged 18–64 years. However, the results of this study show that TEWL has no significant change in dry people, so it may not be comprehensive enough to take TEWL as a single indicator for the determination of skin barrier function. Skin barrier function can be comprehensively measured by combining cuticle water content, oil secretion, and other indicators.

This study compared the differences in microbiota composition between the hands and legs, with results showing that the bacterial diversity (Shannon index, *P* < 0.05) on the back of the hand was lower than that on the lower leg, while the richness (Chao index, *P* < 0.05) was higher. Significant differences in microbial composition were observed at the phylum, genus, and species levels between the two areas. At the phylum level, Firmicutes and Proteobacteria were the dominant bacterial groups, but their abundance distribution exhibited area-specific characteristics. At the genus level, *Cutibacterium* and *Pseudomonas* were markedly enriched on the back hand, whereas *Streptococcus*, *Staphylococcus*, *Thermus*, and *Aeromonas* predominated on the lower leg. At the species level, *Cutibacterium acnes* demonstrated higher abundances on the back hand, contrasting with the pronounced enrichment of *Streptococcus gallolyticus, Thermus scotoductus*, and *Staphylococcus hominis* on the lower leg. β-diversity clustering analyses revealed pronounced segregation of microbiota between the two anatomical areas. These findings suggest that even within similar microenvironments, area-specific physiological traits play a critical role in modulating microbial assembly. In addition, other internal and external factors also play a crucial role in the formation of microbial differences (Dimitriu et al., 2019). For instance: The characteristics of the back of the hand include thin and mobile skin rich in hair follicles and sebaceous glands, which fosters lipid-dependent taxa such as *Cutibacterium acnes* (Edmonds-Wilson et al., 2015). Prolonged environmental exposure amplifies exogenous influences (such as hygiene practices)-hand washing alters microbial diversity (Rosenthal et al., 2013), while frequent moisturizer use reduces *Corynebacterium* but enriches *Streptococcus* (Vindenes et al., 2024). The skin characteristics of the lower leg area include minimal sebum secretion and a thicker stratum corneum, which facilitate the colonization of drought-resistant bacterial genera (*Streptococcus* and *Corynebacterium*) (Findley et al., 2013). It is worth noting that symptoms such as “ichthyosis” and “chicken skin” often occur in the lower leg and arm, which further indicates that the lower leg skin is more prone to dehydration, dryness, tightness than another part (Vahlquist and Torma, 2020). Therefore, further studies and explorations are needed to understand the reasons for the difference in microbial composition between the back of the hand and the lower leg skin. Consequently, the divergence of microbes across different locations is influenced by a combination of host anatomical structure, exposure to the environment, and behavioral aspects (Martinez et al., 2021), which calls for comprehensive models to analyze their individual impacts.

We compared the composition of skin bacteria in the same part (back of hand and lower leg) of healthy and dry people. Dry skin groups exhibited significantly higher microbial diversity (Shannon index, *P* < 0.05) but comparable species richness (Chao index, *P* > 0.05). In addition, our results also showed that *Streptococcus* abundance was markedly elevated in dry skin group (*P* < 0.01), particularly in the lower leg. Meanwhile, *Cutibacterium* exhibited a downward trend, with a 13.03% decrease on the back of the hand (*P* = 0.091) and a 2.782% reduction on the lower leg (*P* = 0.203) in the dry skin group. Byrd et al. (2018) found that *Streptococcus* thrive particularly well in dry skin environments (such as the lower legs and individuals with dry skin). This may be because *Streptococcus* (negatively correlated with hydration, TEWL, and sebum) adapts to low-lipid conditions through dehydration resistance mechanisms (e.g., extracellular polysaccharide synthesis) (Byrd et al., 2018). In contrast, the abundance of *Cutibacterium* (negatively correlated with dryness level) decreases with the reduction of Hydration, TEWL, and sebum. This is because *Cutibacterium* typically inhabits the surface of human skin, particularly in hair follicles and sebaceous gland regions, which feature higher humidity and sebum content that provide essential nutrients and promote its growth (Edmonds-Wilson et al., 2015). The lack of moisture and oil in dry skin is not conducive to its growth (Grice, 2014). Based on the above research findings, we discovered significant differences in hydration and sebum levels between the skin of the back of the hand and the lower leg. There were also notable differences in hydration and sebum levels on the back of the hand between healthy individuals and those with dry skin. However, the trends in microbiome changes between these two groups did not fully align with the skin physiological parameter results. From the perspective of microbial composition, in the healthy population, the dominant bacterial genera (including *Cutibacterium*, *Streptococcus*, *Staphylococcus*, *Thermus*, and *Aeromonas*) exhibited highly significant differences between the back hand and lower leg areas. In the dry skin group, apart from *Streptococcus*, the relative abundances of *Cutibacterium*, *Staphylococcus*, *Thermus*, and *Aeromonas* still showed significant differentiation between areas (See Supplementary Figure 2). However, on the back aspect of the hand, among the top five most abundant bacterial genera, only the abundance of *Streptococcus* showed a significant difference between healthy individuals and those with dry skin. These findings demonstrate that microbial divergence between the back hand and lower leg skin was more pronounced compared to the differences observed between healthy and dry skin populations. This finding aligns with the previous research results of Costello et al. (2009), which pointed out that the differences of microbial populations in different body areas are greater than those among individuals. This phenomenon suggests that the same skin site (e.g., back of hand or lower leg), due to the relative consistency of its intrinsic and extrinsic influencing factors (physiological characteristics, environmental exposure, etc.,) is more likely to develop convergent microbial community features. In contrast, micro environmental heterogeneity (e.g., humidity, sebum secretion) at different areas dominated the significant differentiation of flora composition. The single-network analysis results revealed that the skin microbial networks on the back of the hand and the lower leg were dominated by *Cutibacterium* and *Streptococcus* as core nodes, respectively. The core genera and their interaction networks significantly reflected the micro environmental characteristics specific to skin areas. It further confirms that body areas play a more significant role in microbial composition.

The balance of skin microbiota is an important indicator of human skin health. Within the same host, physiological characteristics of different areas lead to significant differences in microbial composition; Within the same skin area, inter-individual internal and external factors can also alter some microbial compositions. Among them, the skin area has the greatest impact on the microbial community composition. Further studies on dry skin may provide new perspectives for the diagnosis and nursing care of dry skin by analyzing the microbial composition in different body areas (e.g., back of the hands and lower legs). In addition, in the development of future daily chemical products, the balance of bacteria should also be fully considered, and the interaction between microorganisms and the symbiotic environment of the skin should be used to provide gentler and healthier care programs for different skin areas and different skin conditions.

## Data Availability

The original contributions presented in the study are publicly available. This data can be found in here: https://www.ncbi.nlm.nih.gov/, accession number: PRJNA1295745.

## Footnotes

1
http://drive5.com/uparse/

2 Sorceforge https://sourceforge.net/projects/rdp-classifier/, version 2.11

3
https://cloud.majorbio.com/
