# Supplementary material for: Analysis of microbial composition in different dry skin areas of Beijing women
Source: Front Microbiol. 2025 Aug 7;16:1504054. doi: 10.3389/fmicb.2025.1504054 (PMC12369417; doi:10.3389/fmicb.2025.1504054)
Supplement: Supplementary file 1 [file Supplementary_file_1.docx]

Supplementary Material

Supplementary Figure 1


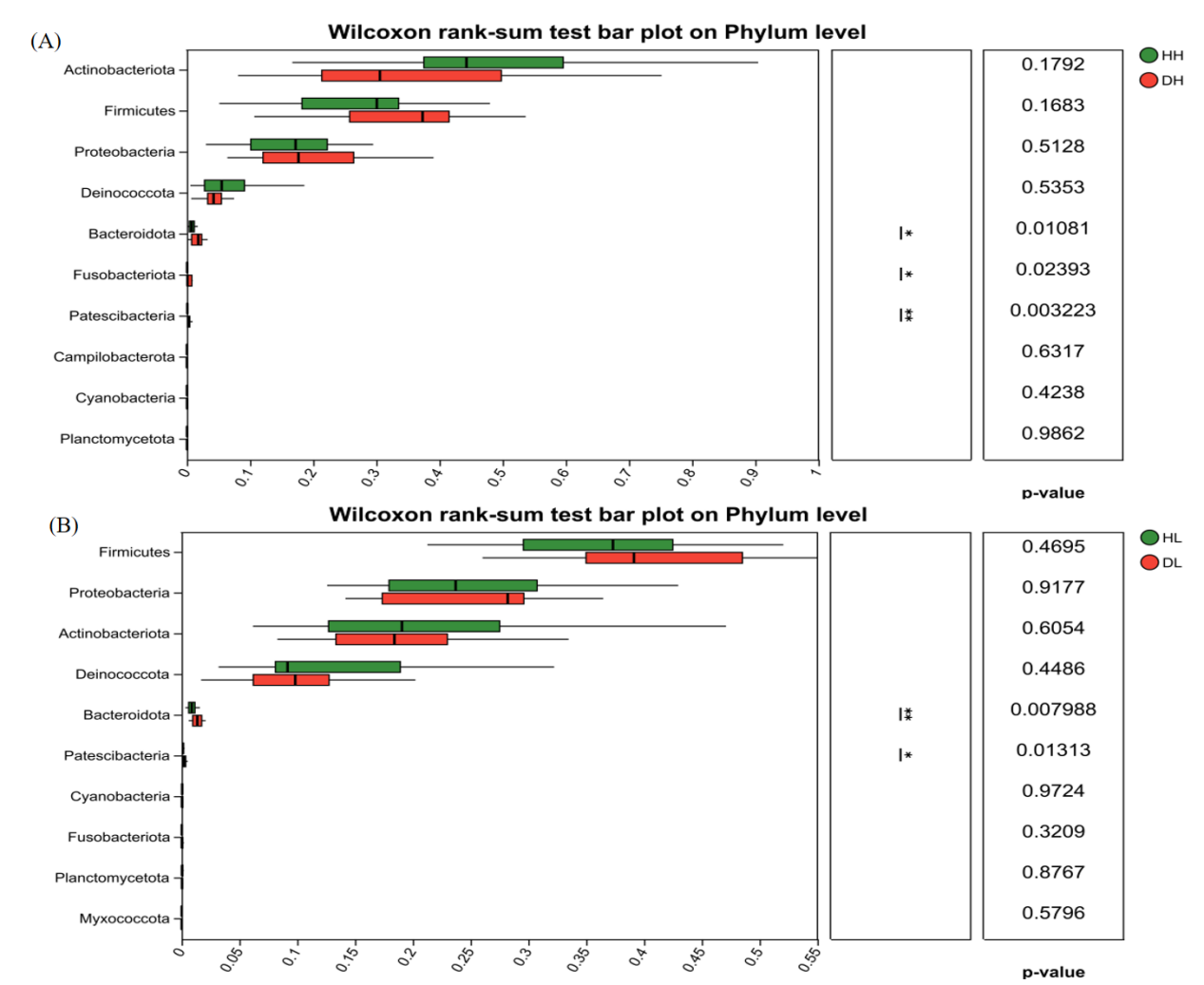
Figure 1: Comparison of bacterial composition at the phylum level between the healthy control group and the dry skin group across different skin areas (A)Differences in the composition of the top 10 phyla in abundance on the back of the hand ; (B) Differences in the composition of the top 10 phyla in abundance on the lower leg; Difference test: Wilcoxon rank sum test was used, where * means 0.01 < *P*≤ 0.05, ** means *P*≤0.01, and *** means *P*≤0.001.

Supplementary Figure 2


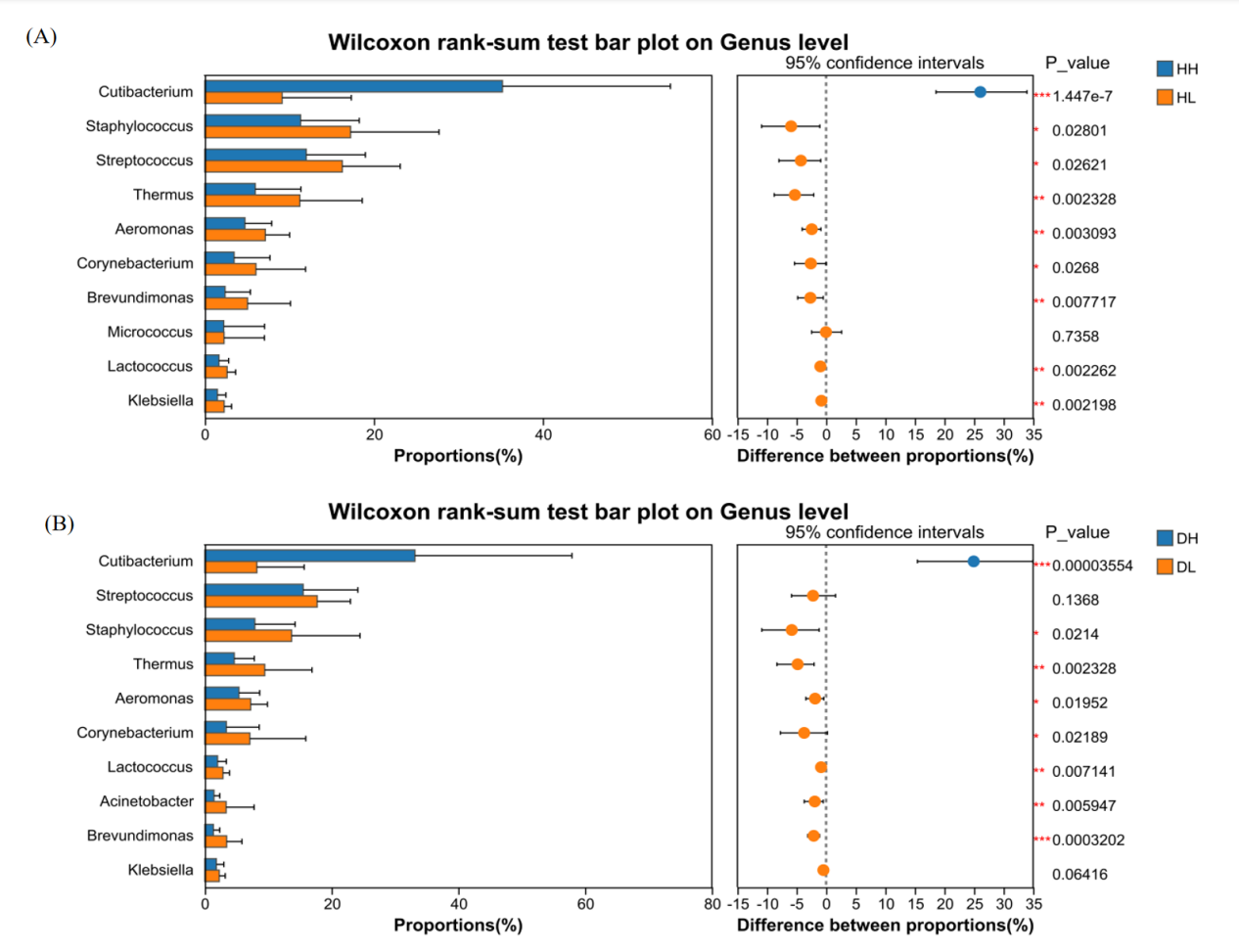
Figure 2: Analysis of compositional differences in bacterial communities. (A) Differential analysis of the top 10 dominant bacterial genera in terms of mean abundance between the back of hands and the lower leg in healthy populations. (B) Differential analysis of the top 10 dominant bacterial genera in terms of mean abundance between the back of hand and the lower leg in populations with xerosis. Difference testing: Wilcoxon rank-sum test was employed, where * means 0.01 < *P*≤ 0.05, ** means *P*≤0.01, and *** means *P*≤0.001.
